# Exploring the live birth rates of women living with HIV in British Columbia, Canada

**DOI:** 10.1371/journal.pone.0211434

**Published:** 2019-02-06

**Authors:** Clara E. Van Ommen, Arianne Y. K. Albert, Micah Piske, Deborah M. Money, Hélène C. F. Cote, Viviane D. Lima, Evelyn J. Maan, Ariane Alimenti, Julianne van Schalkwyk, Neora Pick, Melanie C. M. Murray

**Affiliations:** 1 Faculty of Medicine, University of British Columbia, Vancouver, British Columbia, Canada; 2 Women’s Health Research Institute, British Columbia Women’s Hospital, Vancouver, British Columbia, Canada; 3 Department of Obstetrics and Gynaecology, University of British Columbia, Vancouver, British Columbia, Canada; 4 Faculty of Public Health and Policy, London School of Hygiene and Tropical Medicine, London, United Kingdom; 5 Department of Pathology and Laboratory Medicine, University of British Columbia, Vancouver, British Columbia, Canada; 6 Oak Tree Clinic, British Columbia Women’s Hospital, Vancouver, British Columbia, Canada; 7 British Columbia Centre for Excellence in HIV/AIDS, Vancouver, British Columbia, Canada; 8 Division of AIDS, Department of Medicine, University of British Columbia, Vancouver, British Columbia, Canada; 9 Division of Infectious and Immunologic Diseases, Department of Pediatrics, University of British Columbia, Vancouver, British Columbia, Canada; 10 Division of Infectious Disease, Department of Medicine, University of British Columbia, Vancouver, British Columbia, Canada; Medical University of Warsaw, POLAND

## Abstract

**Objective:**

To evaluate the birth rates of women living with HIV (WLWH) compared to the general population in British Columbia (BC), Canada.

**Methods:**

We retrospectively reviewed clinical and population level surveillance data from 1997 to 2015. Live birth rates from 1997 to 2015 among WLWH aged 15–49 years were compared with those of all BC women. Next, the number of live births among WLWH with a live birth between 1997–2012 and HIV-negative controls matched 1:3 by geocode were compared.

**Results:**

WLWH had a lower birth rate compared to all BC women [31.4 (95%CI, 28.6–34.3) vs. 40.0 (39.3–40.1)/1000 person years]. Stratified by age, WLWH aged 15–24 years had a higher birth rate while WLWH aged 25–49 years had a lower birth rate than BC women (p<0.01). Between 1997 and 2015, birth rates for both populations decreased among women aged 15–24 years, and increased among women aged 25–49 years, most strikingly among WLWH 35–49 years (p<0.01). When comparing WLWH with a live birth to HIV-negative geocode matched controls, WLWH aged 15–24 years (p = 0.03) and aged 25–34 years (p<0.01) had more live births than controls while WLWH aged 35–49 years did not (p = 0.06).

**Conclusions:**

On a population level, WLWH have lower birth rates than the general population. However, this is not observed among WLWH who have ever given birth compared with matched controls, suggesting that sociodemographic factors may play an important role. WLWH are increasingly giving birth in their later reproductive years. Taken together, our data supports the integration of reproductive health and HIV care.

## Introduction

In Canada, the majority of women living with HIV (WLWH) are of reproductive age (15–49 years) [1]. Recent studies have found that most WLWH desire or intend to become pregnant in the future, and indicate that motherhood is important to them [2–5]. Given that many WLWH desire pregnancy, it is important to understand the impact of HIV on the birth rates and reproductive outcomes of WLWH.

The literature on HIV’s impact on birth rates comes primarily from low and middle income countries, showing that HIV infection is associated with lower birth rates [6–11]. Few studies have explored the impact of HIV on birth rates in high income countries, Canada in particular. Furthermore, a number of the aforementioned studies were conducted before the combination antiretroviral therapy (cART) era, or in areas where cART was not readily available, such that WLWH were more likely to be experiencing AIDS defining illnesses. As such, these studies have limited applicability to WLWH currently living in Canada, where 70–82% of people living with HIV, depending on their setting, are receiving any treatment for their HIV and 84–93% of those receiving treatment are virally suppressed [12].

More recent studies, in low and middle income countries [13–16] as well as in the United States [17–19], have observed an increase in pregnancy and birth rates among WLWH after cART became available. In Canada, a study of birth rates of WLWH in Ontario between 2002 and 2010 found that WLWH had birth rates 20% lower than that of the general population [20]. A more recent study of WLWH across Canada found that women who initiated HIV treatment in the post-cART era reported more pregnancies than those who started therapy earlier [21]. Overall, this suggests that women living in high income countries, where cART and effective HIV care is more readily available and accessible, may experience improved birth rates albeit lower than that of the general population.

Given the paucity of data on birth rates of WLWH in developed regions and in Canada during the post-cART era, we first aimed to compare live birth rates and trends over time of WLWH in British Columbia (BC) to those of the general BC population. Secondly, in an effort to further delineate our population, we compared the parity of WLWH who had a live birth in BC to that of HIV-negative women living in the same geocode, restricting to those with a child born during the same year.

## Materials and methods

### Study design and populations

This study was a two-part retrospective review of clinical and population surveillance data. The first part of the study involved an analysis of population data for the entire province of BC (BC Population Analysis). Here, we compared birth rates and trends between WLWH (data source: BC Provincial HIV Perinatal Database) and all BC women (data source: Vital Statistics BC) between 1997 and 2015. This time period reflects the period after cART became available in BC to all pregnant WLWH until the end of currently available comparison data.

Oak Tree Clinic at BC Women’s Hospital in Vancouver, Canada is the provincial referral centre for perinatal care of WLWH, and is involved in the care of >95% of all pregnant WLWH in BC. Since 1994, the clinic has maintained the BC Provincial HIV Perinatal Database with clinical and demographic data collected on all pregnant WLWH in BC, seen either in person at the clinic or discussed remotely. As such, the clinical surveillance data obtained from the BC Provincial HIV Perinatal Database on the number of pregnancies and pregnancy outcomes of WLWH is the most complete approximation of incident pregnancies among WLWH in BC.

The BC Centre for Excellence in HIV/AIDS collects longitudinal data on all persons living with HIV in BC who have ever engaged in treatment, making this dataset the best available estimate of the total number of WLWH who ever received care in BC. Using completely anonymized data from the BC Provincial HIV Perinatal Database (number of pregnancies) and the BC Centre for Excellence on HIV/AIDS (number of WLWH in BC), we determined the live birth rates (births/1000 person years) from 1997 to 2015, among 15–49 year old WLWH in BC. These data were then compared to publicly available anonymized data from Vital Statistics BC on the live birth rates of all BC women aged 15–49 over the same period [22].

The second part of the study involved a secondary use of data, comparing the number of live births among WLWH and HIV-negative women who had previously borne at least one child and were sociodemographically similar (Matched Analysis). The goal of this analysis was to control for sociodemographic factors that may have been influencing the results of the population level analysis, as well as to compare WLWH and HIV-negative controls who had had at least one child. This analysis was done to assess if those WLWH who choose to have children had decreased parity compared to controls. This was achieved through an analysis of BC WLWH and HIV-negative controls, matched approximately 1:3 by geocode. Matching populations by geocode has been shown to increase the similarity of sociodemographic factors [23], though success rests on the premise that the matching process results in adequately similar groups. This matched analysis was a secondary analysis of an anonymized dataset initially designed to study health outcomes among HIV-exposed uninfected (HEU) children born to WLWH in BC between 1990 and 2012 [24]. This original analysis involved all HEU children who: were born to WLWH in BC between January 1, 1990 and December 31, 2012, were HIV-negative themselves, and had personal health numbers on record. For this analysis, we included births from 1997 to 2012 in order to more closely align with the population level analysis and to reflect the post-cART era. The dataset represents >95% of all HEU born to WLWH in BC during the time period. Of note, as this dataset was restricted to mother-HEU pairs, it does not include six WLWH in BC who had a child after 1997 where there was a vertical transmission of HIV. As part of this original analysis, data was also collected for three matched HIV-unexposed uninfected (HUU) children and mother pairs per HEU. Matching was done such that HEU and HUU controls of the same sex were born in the same year, to mothers having the same primary address’ geocode (first three-digit postal code) at the time of the child’s birth.

The anonymized dataset was acquired through the data holder Population Data BC from BC Vital Statistics Agency [25]. The secondary analysis presented herein is that of the children’s mothers, removing repeat live births among women who had more than one child during the study period, along with their matched controls. This time period was chosen because it encompassed all available data on births to WLWH in BC at the time the data request was initiated. Ethical approval was obtained from the BC Women and Children’s Ethics board (H16-02345-A001).

### Statistical analysis

#### BC population analysis

Statistical analysis was conducted using R [26]. Birth rates are expressed as births/1000 person years and as age adjusted risk ratios (RR). Live birth rates between the groups were also compared after stratifying into the following age groups: 15–24, 25–34, and 35–49 years. Negative binomial regression was used to compare the live birth rates between WLWH and all BC women for the three age groups between 1997 and 2015.

#### Matched analysis

The number of unique live births (mothers were included only once if they had more than one child over the study period), to WLWH and HIV-negative controls from 1997 to 2012 was determined as an entire group (15–49 years), and in three age stratified groups (15–24, 25–34, and 35–49 years). Women with missing live birth data were also excluded, thus perfect 1:3 matching was not observed. The average number of births/woman in that time period was also calculated and compared between the groups using the Wilcoxon rank sum test. It should be noted that during the age stratification process the geocode matching of the women was partially lost. Despite this, we believe that the HIV-negative control women still represent a good estimate of being sociodemographically similar to the WLWH. Herein, our analysis of this data is limited by two primary factors. First, as stated above, during the age stratification process the geocode matching was partially lost. Second, we are only considering WLWH and control women who have already had at least one child, thus biasing our population towards women who are potentially more engaged in care and who desire to bear children.

## Results

### BC population level analysis (1997–2015)

#### Birth rates of WLWH vs. all BC women

In BC, between 1997 and 2015, there were a total of 456 live births among 14,539 person-years of follow up for WLWH, for an average of 24 births/year. There were 811,213 live births among 20,300,406 person-years of follow up for all BC women over the same period, for an average 42,695 births/year. WLWH represented approximately 0.05% of all live births in BC over this time period.

Overall, WLWH had a significantly lower crude birth rate compared to all BC women [31.4/1000 person years (95%CI = 28.6–34.3) vs. 40.0/1000 person years (95%CI = 39.3–40.1)], with a significantly lower age-adjusted risk ratio of (RR = 0.85, 95%CI = 0.75–0.95, p<0.01).

#### Birth rates of WLWH vs. all BC women by age group

**Fig 1:**
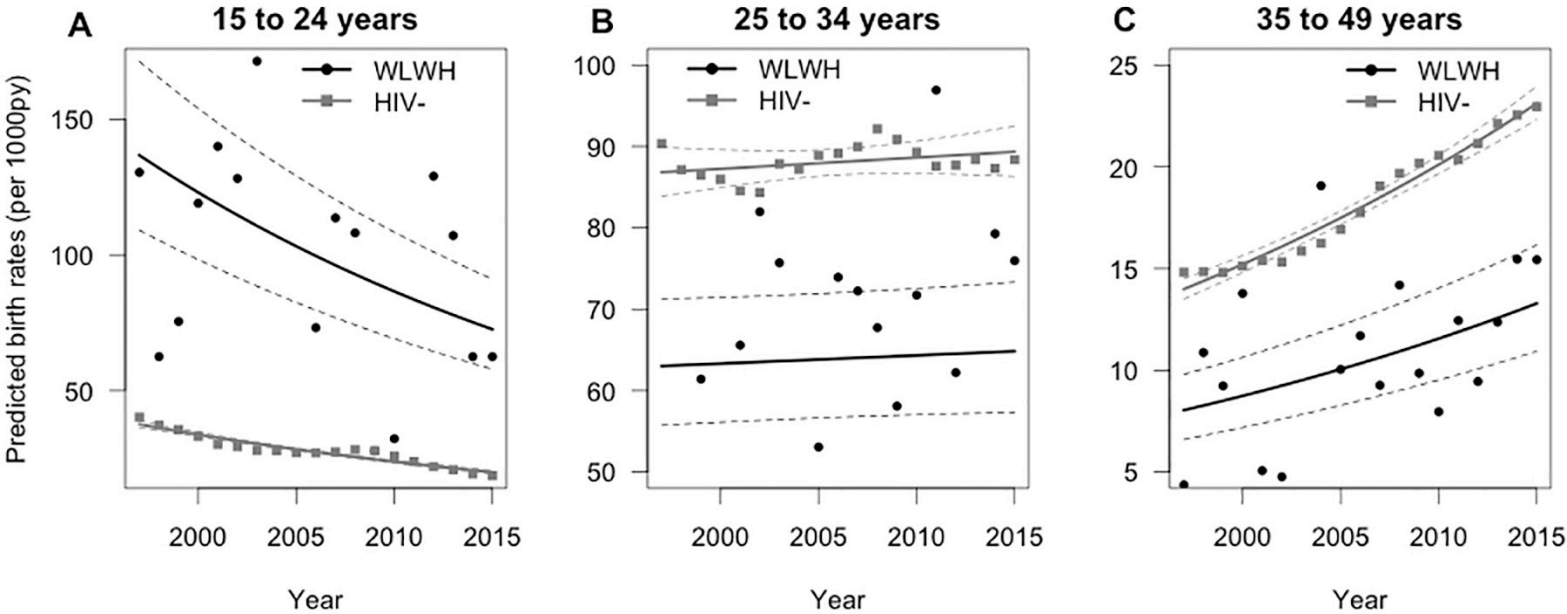
BC predicted live birth rates over time by HIV status and age group.

Overall, there was a significant interaction between HIV status and age group (p<0.0001) adjusting for year. To fully explore this interaction term, we examined the relationships within subgroups by age. When examined by age group, WLWH aged 15–24 years had a higher live birth rate than BC women (p<0.01) whereas WLWH aged 25–49 years had lower live birth rates than BC women (p<0.01) (Fig 1) (Table 1). For both WLWH and BC women aged 15–24 years, birth rates decreased over time (p<0.01). In contrast, for WLWH and BC women aged 25–49 years, birth rates increased over time (p<0.01) (Fig 1) (Table 1).

**Table 1:** Number of births and birth rates by age group and HIV status for 1997, 2006 and 2015 in BC.

	15 to 24 years			25 to 34 years			35 to 49 years		
	Number of births	Person years	Births/1000 person years (95%CI)	Number of births	Person years	Births/1000 person years (95%CI)	Number of births	Person years	Births/1000 person years (95%CI)
**WLWH in BC**									
1997	6	46	137 (109–171)	9	194	63 (56–71)	1	229	8 (7–10)
2006	3	41	100 (80–125)	19	257	64 (57–72)	6	513	10 (9 to 13)
2015	2	32	73 (58–91)	12	158	65 (57–73)	10	648	13 (11 to 16)
**BC Women**									
1997	10 252	254 901	38 (36–39)	27 847	308 201	87 (84–90)	7205	486 175	14 (13–14)
2006	7533	278 628	27 (27–28)	24 608	276 060	88 (86–90)	8986	506 223	18 (18–18)
2015	5361	287 473	20 (19–21)	28 569	323 261	89 (86–92)	10 916	475 585	23 (22–24)

**Fig 2:**
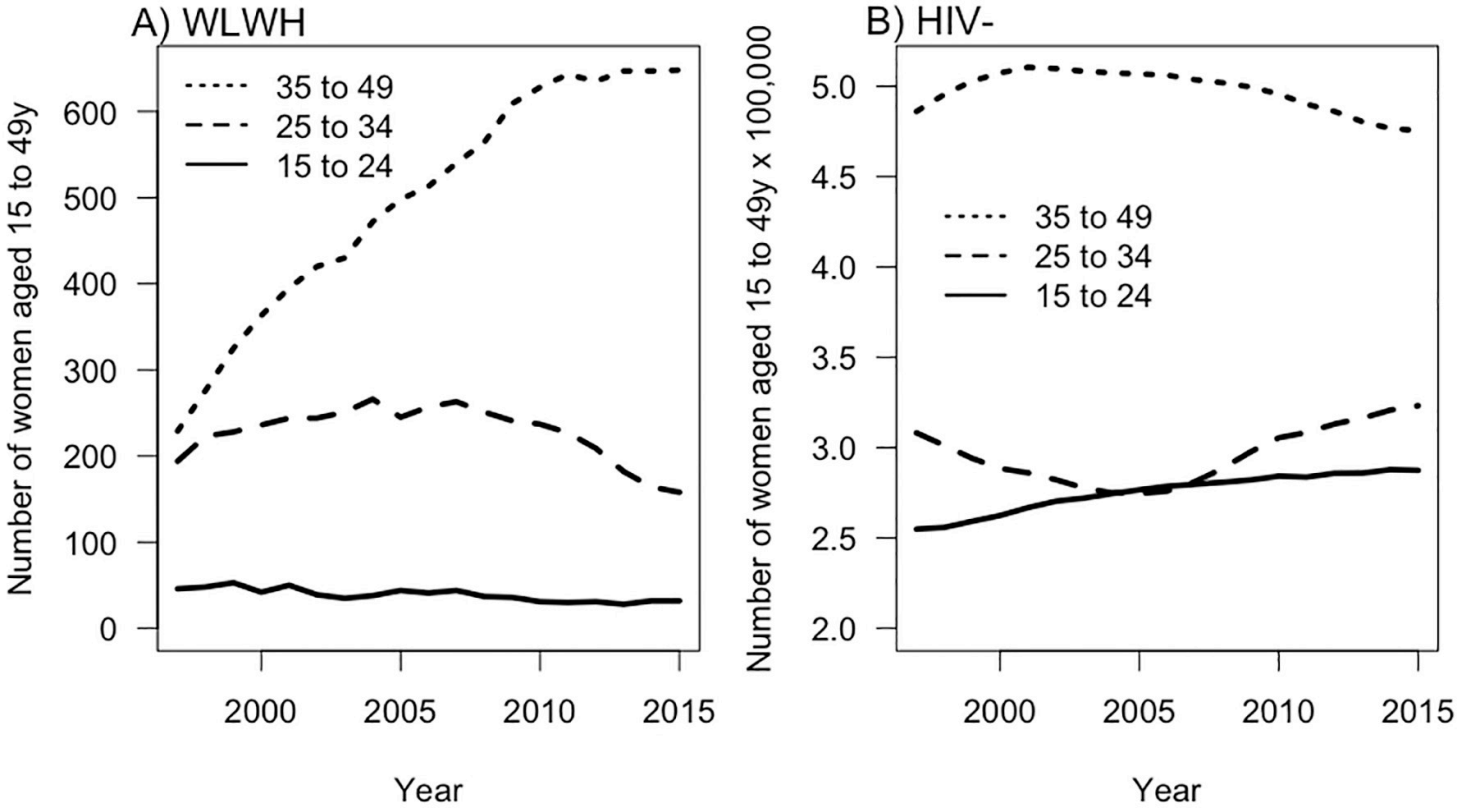
Proportion of WLWH and BC women by age group over time.

While live birth rates as described above are the optimal way to consider the trends we observed over time, we also noted a trend in the proportion of women in each age group and the proportion of births to each age group. We demonstrate this using three representative years from the beginning, middle and end of our study time period in order to illustrate the observed trends (Table 1). Over the 19-year time span, the proportion of WLWH in each age group changed. As a raw count of women, the number of WLWH aged 35–49 years increased (Fig 2). This is in contrast to the BC population, where the number of women in each age group remained relatively constant over the time period (Fig 2). In both groups, the proportion of all births that were to women aged 35–49 years increased over time although this increase was most apparent within WLWH. For example, in 1997, 6.3% of births to WLWH were to women aged 35–49 years. By 2015 this had changed considerably such that 41.7% of births to WLWH were to women aged 35–49 years (Table 1). In 1997, among all BC women, 15.9% of births were to women aged 35–49 years, and this increased to 24.3% of births in 2015 (Table 1).

### Matched analysis (1997–2012)

The matched analysis from 1997–2012 included 270 WLWH and 871 HIV-negative controls. There was no significant difference in the mean age of the women between the two groups. Within this cohort there were 669 live births to WLWH and 1577 to HIV-negative controls. Considering women of all ages, WLWH had more live births per woman than the HIV-negative controls (p<0.01). Among women aged 15–24 years (p = 0.03) and aged 25–34 years (p<0.01), WLWH had more live births per woman than HIV negative controls. Among women aged 35–49 years, there was no significant difference in the number of live births per woman between WLWH and HIV-negative controls (p = 0.06) (Table 2).

**Table 2:** Number of live births to WLWH and geocode matched^1^ HIV-negative controls from 1997–2012.

	All ages (15–49 years)	15–24 years	25–34 years	35–49 years
**WLWH (N = 270)**				
**Total live births**	669	79	412	178
**Mean live births/woman, mean (95%CI)**	2.49 (2.31–2.67)	1.88 (1.65–2.20)	2.62 (2.38–2.87)	2.58 (2.19–2.96)
**HIV negative controls (N = 871)**				
**Total live births**	1577	220	886	471
**Mean live births/woman, mean (95%CI)**	1.82 (1.75–1.88)	1.52 (1.39–1.64))	1.76 (1.68–1.84)	2.11 (1.96–2.26)
**P- value**	**<0.01**	**0.03**	**<0.01**	0.06

^1^Women with missing live birth data were excluded, thus perfect 1:3 matching is not achieved.

## Discussion

Our study shows that in an era of good access to effective cART, WLWH in BC still experience an overall birth rate lower than the general population. These results align with other studies examining the live birth rates of WLWH. A study of WLWH aged 15–49 in Ontario observed a live birth rate of 35.3 vs. 44.4 per 1000 woman years in the general Ontario population from 2002–2010 [20]. These Ontario birth rates reported for WLWH and the general population were both higher than those observed in our BC study. This may be related to differences in the ethnic origins and sociodemographic makeup of WLWH in these two provinces. Ontario has a larger proportion of the population with ancestry in Africa and the Caribbean than BC does [27]. Indeed the Ontario study reported a higher birth rate among WLWH with African or Caribbean origins compared to others [20]. In contrast, a recent study on the birth rates of WLWH in the United States found significantly lower birth rates among WLWH compared to HIV-negative women at high risk for HIV acquisition from 1994–2001, but no difference during the 2002–2012 period. This latter observation may be at least partially explained by the fact that the control population for this study consisted of high-risk HIV-negative women in whom substance use and/or increased illness burden may decrease birth rates relative to the general population [18].

Overall, it appears that since the use of cART became widespread, WLWH around the world have experienced an increase in birth rates [16,18]. Despite this, as a group, WLWH in BC still experience lower birth rates compared to the general population. The reasons behind this are likely multifactorial and may include social circumstances, co-morbid health conditions, changes in reproductive health, uncertainty associated with living with a chronic disease, and stigma against childbearing for WLWH [21,28–31].

We also observed clear age-related differences in birth rates with younger WLWH having a higher birth rate than the general population while the reverse was true for older WLWH. The higher birth rate observed in younger WLWH may be related to lower rates of birth control use and higher rates of unprotected intercourse, which could be associated with both HIV acquisition and pregnancy. For example, a recent study of WLWH across Canada observed that younger WLWH were more likely to experience an unintended pregnancy [21]. There is also wealth of knowledge that perinataly infected youth are less engaged in care and virally suppressed [32], hence they may access health services less, including reproductive health and contraception education. This may suggest that special attention should be given to young women aged <30 years, due to both their low rate of HIV suppression, and higher rates of unintended pregnancies.

In regards to older WLWH, we observed the same trend in changing birth rates as the general population, with a delay in child bearing toward later life. Importantly, it appears that older WLWH are increasingly likely to have a pregnancy in their later reproductive years. This is despite some literature suggesting that WLWH have a more rapidly diminishing ovarian reserve [33]. Nevertheless, this pattern is important, as later childbearing is associated with greater morbidity for mother and infant, regardless of HIV status [34]. An awareness of this general trend could help guide clinicians in informing their patients about the unique risks and benefits associated with delaying child bearing as a WLWH.

Interestingly, when we compared WLWH and geocode matched (assumed socio-demographically similar) HIV-negative women with proven parity, we observed that WLWH experience as many or more live births as their HIV-negative peers. This supports the argument that additional factors may preclude women from childbearing altogether, and may modulate the differences seen when comparing WLWH to the general population. This may also be due to the fact that nearly all of the WLWH included in this study received care at Oak Tree Clinic, where they received integrated HIV and reproductive care, and as such, may feel more comfortable becoming pregnant. However, this interpretation is limited given that the factors that may be influencing WLWH in their decision making regarding childbearing (ie. their first pregnancy), may also be influencing further pregnancies. Taken together, this finding suggests that WLWH who chose to have at least one child tend to have as many or more live births as HIV-negative women of presumed similar socio-demographic status.

Strengths of our study include the population level data reliably collected for WLWH in our province. Our study is also the first to examine birth rates at the population level of WLWH in BC. However, we are limited by the small number of WLWH included when compared to the larger BC population. Further, we did not have data available to further investigate predictors of childbearing for WLWH in our province, nor data on the pregnancy intent of these women. Finally, our matched analysis was based on WLWH who had HEU children, so it did not include birth rates for the 2% of women who had an HIV-infected child (and no HEU child) in the time period. It also only included women with at least one child, so does not take into consideration women who chose not to child bear, women who chose to terminate their pregnancies, or who could not have children. Finally, we were only able to attain partial geocode matching, though we believe that this is still a good representation of sociodemographic similarity, we are limited in our interpretation in this regard.

## Conclusions

In conclusion, we observed that, as a group, WLWH experience a lower birth rate than the general population, but when sociodemographically matched this difference did not persist suggesting this is not a biologic phenomenon but likely related to social circumstances. Of note, on a population level, WLWH are increasingly likely to bear children later in life, something that has implications for the care of WLWH and their reproductive planning. The results of this study are an important step toward further understanding the reproductive health trends of WLWH, especially now that WLWH can have pregnancies with little or no risk of vertically transmitting HIV to their child if engaged in care and appropriately treated. This study provides additional evidence that the care of WLWH who are within their reproductive years must include appropriate reproductive care alongside their HIV care, and counselling to explore their pregnancy plans and desires. Ultimately, this supports the need to integrate reproductive health care into a comprehensive HIV care program for every woman living with HIV and ensure pregnancy planning is included in HIV care [35].
